# Low incidence of choroidal neovascularization following subthreshold diode micropulse laser (SDM) in high-risk AMD

**DOI:** 10.1371/journal.pone.0202097

**Published:** 2018-08-23

**Authors:** Jeffrey K. Luttrull, Stephen H. Sinclair, Solly Elmann, Bert M. Glaser

**Affiliations:** 1 Private practice, Ventura, California; 2 Drexel University School of Medicine, Philadelphia, Pennsylvania; 3 National Retina Institute, Towson, Maryland; Roskamp Institute, UNITED STATES

## Abstract

**Purpose:**

To determine the incidence of new choroidal neovascularization (CNV) in eyes with dry age-related macular degeneration (AMD) following subthreshold diode micropulse laser (SDM).

**Method:**

In an observational retrospective cohort study, the records of all patients active in the electronic medical records database were reviewed to identify eyes with dry AMD treated with SDM. Identified eyes were classified by simplified AREDS categories, and analyzed for the primary endpoint of new CNV after treatment.

**Results:**

The EMR revealed SDM was offered to 373/392 (95%) patients with dry AMD and elected by 363/373 (97%) between 2008–2017. Follow up was available for 354/363 patients (547 eyes, 98%) (range 6–108 mos., avg. 22). CNV risk factors included age (median 84 years, 67% > 80); reticular pseudodrusen (214 eyes, 39%); AREDS category (78% category 3 and 4); and fellow eye CNV (128 eyes, 23%). New CNV developed in 9/547 eyes (1.6%, annualized rate 0.87%). Visual acuity was unchanged. There were no adverse treatment effects.

**Summary:**

In a review of a large group of eyes with exceptionally high-risk AMD, SDM was followed by a very low incidence of new CNV. If confirmed by further study, SDM would offer a new and highly effective treatment to reduce the risk of vision loss from AMD.

## Introduction

Age-related macular degeneration (AMD) is the principal cause of irreversible vision loss in persons over 50 years of age worldwide. [1] The estimated global prevalence of AMD in 2020 is projected to be 196 million, growing to 288 million by 2040. [1, 2] In a 2004 estimate, 7.3 million in the US had high-risk AMD, with 1.75 million suffering severe visual loss due to advanced AMD, consisting of subfoveal age-related geographic atrophy (ARGA, 10% of cases), and choroidal neovascularization (CNV, 90% of cases). Advanced AMD is estimated to account for 46% of all cases of visual loss to 20/200 or worse in the US. [2–14] Currently, the only therapeutic measure known to reduce the risk of advanced AMD is oral antioxidant therapy, which may reduce the risk of severe visual loss (mostly due to development of CNV) by as much as 25% over six years. [9, 13]

Senescent cell damage is due to an accumulation of intracellular protein abnormalities that may lead to various types of cellular dysfunction, including altered cytokine expression and response, apoptosis, and cell death. [14–19] In the retina, this may result in disturbances of autoregulation and chronic inflammation that promote vasoproliferation, and choroidal neovascularization (CNV). Heat Shock Proteins (HSPs) have manifold functions improving cell survival and function in response to acute sublethal cellular stressors. [16, 20–40] Activation of retinal pigment epithelial (RPE) HSPs by retinal laser exposure, including low-intensity / high-density subthreshold diode micropulse laser (SDM), has been demonstrated both *in vitro* and *in vivo*, and is postulated to be the initiating event in the therapeutic mechanism of action of retinal laser treatment. [21, 25, 29, 31, 35–46]

SDM has been found to improve retinal and visual function in eyes with dry AMD. [41] In this report we examine the incidence of choroidal neovascularization (CNV) in eyes with dry AMD following SDM.

## Methods

This observational retrospective cohort study was performed following approval by the Western Investigational Review Board (IRB). It complied with the Health Insurance Portability and Accountability Act of 1996, and the tenets of the Declaration of Helsinki. As a retrospective review of EMR, prior written patient consent was neither obtained, nor required by the approving IRB. All data was anonymized prior to analysis.

### Study population

The records of all patients active in an EMR (JKL) with AMD of AREDS category 2 or greater in at least one eye were identified. The performance of SDM, treatment dates and, and laser parameters were recorded. Eyes with other causes of CNV, such as ocular histoplasmosis, polypoidal choroidal vasculopathy, macular telangiectasis, prior macular photocoagulation, and degenerative myopia were excluded. However, eyes with dry AMD treated by SDM for other primary diagnoses, such as concurrent branch retinal vein occlusion or diabetic macular edema were included, as these co-morbidities have little effect on the risk of CNV from AMD, and the treatment (panmacular SDM) was identical. [47]

For eyes with dry AMD as the primary treatment indication, treatment was offered based on the following considerations: 1) the progressive nature of the disease and risk of visual loss absent effective treatment; 2) absence of effective treatment beyond AREDS supplements; 3) the safety of SDM, known to have only therapeutic effects in all reported applications in over 18 years of clinical use, and; 4) the finding of improved retinal and visual function following SDM in AMD, suggesting treatment might slow disease progression. [15–47] Initially, SDM treatment of AMD was largely incidental to treatment for other primary indications, such as DME. Improved understanding of the mechanism of SDM and the ability to detect, document and monitor treatment effects lead to increased offering of SDM for AMD as a primary treatment indication. [33, 41–43] Return of functional testing indices to pre-treatment baselines, generally 6–8 months following treatment, served as a signal for retreatment.[41] Treatment was offered for a minimum of AREDS category 2 AMD in at least one eye. Fellow eyes with of AREDS category 1 or greater were also treated. Fellow eyes without AMD were not treated.

To investigate possible selection bias, all study eligible eyes in the EMR not treated by SDM were also identified and recorded. These included patients who declined treatment, and those for whom treatment was not recommended due to such non-ophthalmic factors as dementia, hospice care, and/or anticipated relocation from the practice area. All patients had infrared, red-free and autofluorescence high-resolution fundus photography, spectral-domain optical coherence tomography (OCT) before and after treatment and at regular intervals (generally every 6 months) thereafter; and had at least 6 months of postoperative follow up. Fundus fluorescein angiography (FFA) and indocyanine green angiography (ICGA) were performed if CNV was suspected, based on clinical findings and OCT.

### Study endpoint

The primary study endpoint was the incidence of new CNV following SDM.

### AMD classification

All eyes were categorized according to the simplified AREDS scale based on fundus photographs and spectral-domain optical coherence tomography (OCT), upon agreement of 2 readers (SE and JKL). [6,8] The diagnosis of new CNV was confirmed by FFA and ICGA. The presence of reticular pseudodrusen (RPD) was also recorded, but not subtyped. As RPD were not recognized in the AREDS and have been subsequently associated with an increased risk of advanced AMD, eyes with RPD were classified in this study as either AREDS category 3 or 4, depending upon other coincident findings. [6, 8]

### SDM treatment

Following informed consent and pupillary dilation, topical proparacaine was applied to the cornea. A Mainster macular contact lens (Ocular Instruments, Mentor, Ohio, magnification factor 1.05x) was placed on the cornea with the aid of a coupling agent. Under minimum slit-lamp illumination, the entire posterior retina circumscribed by the posterior vascular arcades, including the fovea, was “painted” with approximately 1100–1800 confluent spot applications of SDM (“panmacular” treatment). [41] Fixed laser parameters used in all eyes were 810nm wavelength, 200um aerial spot size, 5% duty cycle; 1.43 Watt power and 0.15 second duration (Oculight SLx, Iris Medical / Iridex Corp, Mountain View, California). Thus, both the treatment technique and laser parameters used were identical in all eyes.

### Data collected

Clinical data recorded included eye laterality, sex, age, Snellen visual acuity, smoking status, presence of systemic hypertension, use of AREDS supplements, number of SDM treatment sessions, months of follow up from the initial SDM treatment to the most recent visit, AREDS category of both the treated and fellow eye, the occurrence of new CNV following SDM, and the number of months following SDM at which any new CNV occurred. [7, 8, 35] The presence of CNV was defined by either of the presence of subretinal fluid and/or macular edema by OCT associated with other photographic findings of AMD, confirmed by subretinal leakage from the CNV on FFA and/or late hyperfluorescence by indocyanine green angiography. [1–10] Once identified, eyes developing new CNV were managed in the customary fashion with intravitreal anti-vascular endothelial growth factor (VEGF) inhibitors.

### Statistical analysis

Study data were anonymized prior to statistical analysis. Frequencies, means, and medians were calculated to summarize the data. The models included fixed eye effects and a random patient intercept to account for inter-eye correlation. Additional hierarchical linear models to explore the association between the difference (post- minus pre-treatment) and pre-treatment values were also performed. Statistical analyses were performed using SAS 9.4 (SAS Institute; Cary, NC).

## Results

### Demographics

392 patients (590 eyes) with dry AMD of AREDS category 2 or greater in at least one eye were identified in the EMR, containing records of all patient visits between April of 2014 through May of 2017. 373 (95%) were offered SDM. 19 patients (27 eyes, 5%) were not offered treatment due to non-ophthalmic considerations. 10 patients (16 eyes, 3%) declined treatment. Thus, 354 patients (547 eyes, 93%) with dry AMD of AREDS Category 2 or greater in at least one eye were identified as having received SDM treatment over an 8.5-year period, between May of 2008 through December of 2016. 9 treated patients (12 eyes, 2% of treated eyes) were lost to follow up before their first post-treatment examination. All patients lost to follow up were successfully contacted to determine the reason for failure to return to the clinic. These included death or illness (5), change in medical insurance (3), or relocation (1). None reported a poor treatment outcome. (Table 1)

**Table 1:** Descriptive statistics of patient and eye level variables by CNVM.

Variable	Value	CNVM		
		No	Yes	Total
**Patients, N (%)**		345 (97.5)	9 (2.5)	354
**Sex**	Female	201 (56.7)	6 (66.7)	207 (58.5)
	Male	144 (40.6)	3 (33.3)	147 (41.5)
**Age**		82.6 (9.0)	81.0 (6.1)	82.5 (8.9)
**Age**	< 80	115 (33.3)	4 (44.4)	119 (34)
	80 +	230 (66.7)	5 (55.6)	235 (66)
**Hypertension**	No	142 (41.3)	7 (77.8)	149 (42.0)
	Yes	202 (58.7)	2 (22.2)	205 (58.0)
**Smoker**	No	325 (94.2)	9 (100.0)	334 (94.4)
	Yes	20 (5.8)	0 (0.0)	20 (5.6)
				
**All Eyes, N (%)**		538 (98.4)	9 (1.6)	547
**Eye**	OS	270 (50.2)	5 (55.6)	275 (50.3)
	OD	268 (49.8)	4 (44.4)	272 (49.7)
**Follow-up time, months**		21.7 (11.8)	15.4 (8.8)	21.6 (11.8)
**Pre-SDM LogMAR**		0.4 (0.3)	0.2 (0.2)	0.4 (0.3)
**Post-SDM LogMAR**		0.4 (0.4)	0.2 (0.1)	0.4 (0.4)
**SDM Treatments**	1	100 (18.6)	0 (0.0)	100 (18.3)
	2	149 (27.7)	4 (44.4)	153 (28.0)
	3	145 (27.0)	4 (44.4)	149 (27.2)
	4	115 (21.4)	1 (11.1)	116 (21.2)
	5	25 (4.6)	0 (0.0)	25 (4.6)
	6	4 (0.7)	0 (0.0)	4 (0.7)
**AREDS Class**	1	9 (1.7)	0 (0.0)	9 (1.6)
	2	107 (19.9)	0 (0.0)	107 (19.6)
	3	278 (51.7)	7 (77.3)	284 (51.9)
	4	145 (27.0)	2 (22.2)	147 (26.9)
**Reticular Pseudodrusen**	No	332 (61.7)	1 (11.1)	333 (60.9)
	Yes	206 (38.3)	8 (88.9)	214 (39.1)
**Fellow Eye AREDS Class**	1	9 (1.7)	0 (0.0)	9 (1.6)
	2	86 (16.0)	0 (0.0)	86 (15.8)
	3	224 (41.7)	3 (33.3)	227 (41.6)
	4	218 (40.6)	6 (66.7)	224 (41.0)
**Fellow Eye Pre-SDM CNVM**	No	415 (77.1)	4 (44.4)	419 (76.6)
	Yes	123 (22.9)	5 (55.6)	128 (23.4)

Descriptive statistics by new CNV events for patient-level and eye-level covariates. Significance testing was not performed due to the very low number of events. N = number. CNV = choroidal neovascularization. SDM = panmacular low-intensity / high-density subthreshold diode micropulse laser. AREDS = age-related eye disease study. AMD = age-related macular degeneration. VA = visual acuity.

The 354 patients included for study were aged 61–103 years (avg. 82, median 84), with 67% 80 years of age or more. 207 were female and 147 male. Post treatment follow up ranged 6–108 months (avg. 22), with 547 eyes followed 6 months or more; 441 eyes 12 months or more; and 237 eyes followed 24 months or more. All patients reported use of AREDS nutritional supplements appropriate to their smoking status throughout the study period.^7^ No eligible eyes were excluded from study.

SDM treatment was applied on average every 8 months, with the average number 2.7 treatments per eye, ranging from 1–6.

Snellen visual acuities were unchanged. There were no adverse treatment effects. (Table 1)

### CNV incidence after SDM treatment

**Fig 1:**
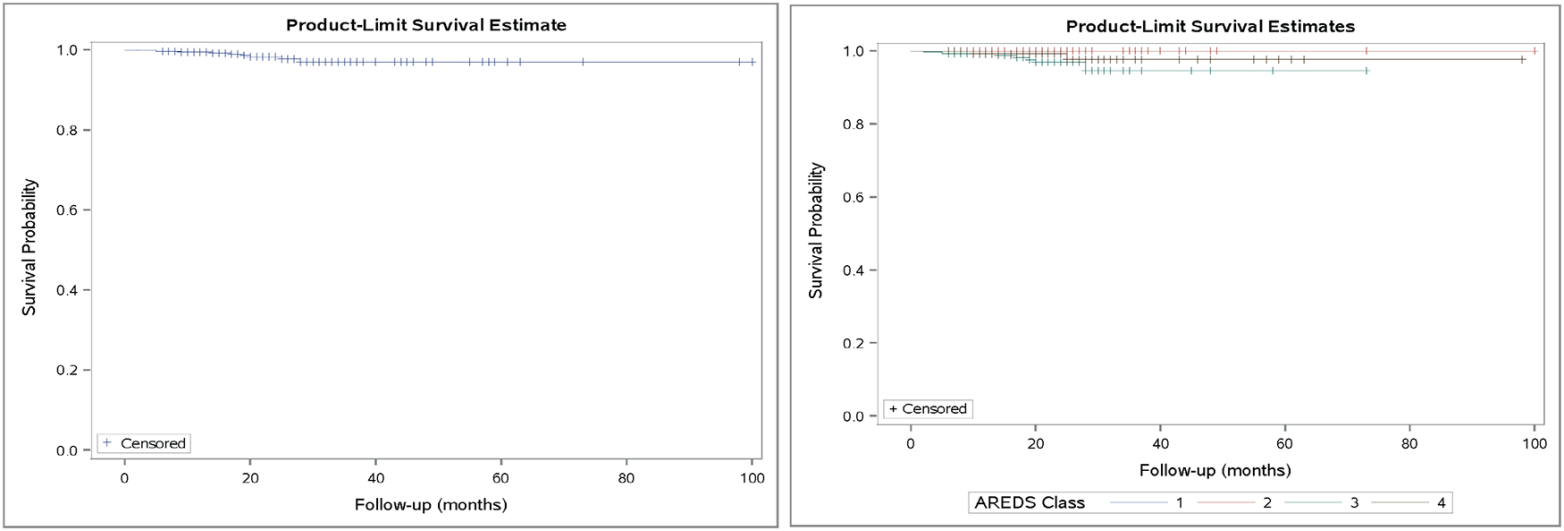
Kaplan-Meier plots showing the event probability across follow-up time overall (left) and by ARED category. The steps down on the curve indicate the CNV cases, while the notches indicate censorship, when follow-up ended for non-cases.

**Fig 2:**
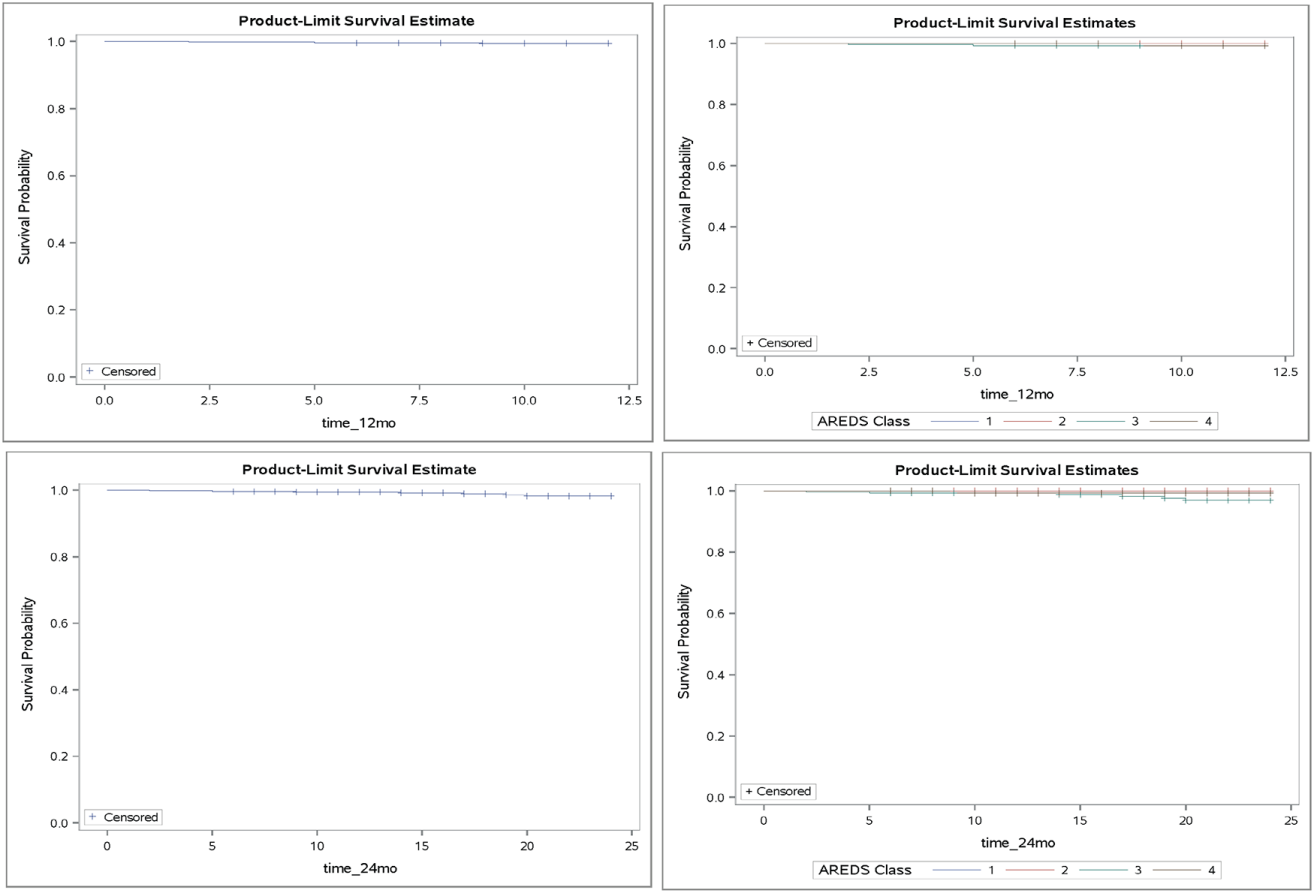
Kaplan-Meier plots showing the event probability across follow-up time overall (left) and by AREDS categories (right); for 12 months (top) and 24 months (bottom) of follow up. The steps down on the curve indicate the CNV cases, while the notches indicate censorship, when follow-up ended for non-cases.

Overall, 9 of 547 eyes with dry AMD treated by SDM developed new CNV (1.64%; annualized rate 0.87%), 2–28 months (avg. 15) following treatment. Of eyes with dry AMD in both eyes prior to treatment, 3/333 developed CNV after SDM (0.90%; annualized rate 0.49%). Of treated eyes presenting with a pre-existing CNV in the fellow eye, 5/128 developed CNV after SDM (3.9%; annualized rate 2.1%). (Tables 1–5) (Figs 1 and 2).

**Table 2:** Rates of progression by age.

Age	Number of Progressions	Person-Eye Months	Rate (per 1000 Person-Eye Months)
**All**	9	11799	0.763
**55–69**	0	1018	0.000
**70–79**	4	2884	1.387
**80–89**	5	5345	0.935
**90 +**	0	2552	0.000
**55–79**	4	3902	1.025
**80 +**	5	7897	0.633

Age-specific rates of progression using two different age groupings. There are no progressions in the youngest or oldest eyes. The second grouping shows that the rate of progression is higher for those under 80 compared to those 80 or over. In this study, the incidence rate ratio is 1.619 comparing those younger than 80 to those 80 or older.

**Table 3:** Univariate Cox regression models using sandwich estimator to account for inter-eye correlation.

Covariate		HR (95% CI)	p-value
**Sex:**	**Female**	1.78 (0.48, 6.64)	0.39
	**Male**	REF	
**Age**		0.98 (0.94, 1.02)	0.24
**Pre-SDM LogMAR**		0.06 (0.00, 1.91)	0.11
**HTN**		0.21 (0.04, 0.98)	0.048
**RPD**		14.62 (1.82, 117.65)	0.01
**Fellow Eye Pre-SDM CNVM**		3.64 (0.98, 13.59)	0.054

Univariate Cox regression models that estimate the effect of covariates on the hazard of progression. A sandwich estimator to obtain robust standard errors was used in order to counteract possible inter-eye correlation. There we too few new CNV events to permit analysis by AREDS category. Systemic hypertension, reticular pseudodrusen, and fellow-eye CNV all predisposed to development of a new CNV.

**Table 4:** Characteristics of treated eyes with dry AMD developing choroidal neovascularization following SDM.

Patient	Age	Sex	AREDS Class treated eye	AREDS class fellow eye	CNV Fellow eye	Entry VA	HBP	Smoke	RPD	No. SDM	Mos. to new CNV
1	87	M	3	4	+	20/30				3	17
2	83	F	3	4	+	20/25	+		+	1	2
3	88	F	3	4	+	20/40			+	1	4
4	73	M	3	3		20/25			+	2	24
5	73	M	4	4	+	20/60			+	2	9
6	82	F	3	3		20/25			+	2	15
7	77	F	3	4	+	20/30			+	2	28
8	82	F	3	3		20/30			+	2	14
9	89	M	4	4		20/60	+		+	4	25
Avg.	82		3.2	3.7	5/9	20/36	2/9	0/9	8/9	2	15

Avg = average. M = male. F = female. AREDS = age-related eye disease study. CNV = choroidal neovascularization. VA = Snellen visual acuity. HBP = high blood pressure. RPD = reticular pseudo drusen. No. = number. Mos. = months.

**Table 5:** Non-age adjusted anatomic subgroup analysis of incidence of new choroidal neovascularization observed in this study, compared to ARED and CATT studies.

AREDS Category*	RPD	No. Eyes	Avg. Age	Avg. FU	Observed new CNV/Annualized Rate	Expected new CNV**/Annualized Rate
1 and 2, treated eye	-	116	77.8	23	0	< 1%
3, treated eye Overall	NA	284	81.5	19.5	7 / 1.5%	19 / 4%
3, treated eye	-	139	79	20.2	1 / 0.4%	NA
3, treated eye	+	146	84	18.9	6 / 2.6%	19 / 8%
4, treated eye Overall	NA	147	87.3	24.7	2 / 0.7%	14 / 5%
4, treated eye	-	79	87.1	27.7	0	NA
4, treated eye	+	68	87.5	21.2	2 / 1.7%	12 / 10%
If fellow eye 4 Overall	NA	224	84.9	23.6	6 / 1.4%	43/ 10%
If Fellow eye 4	-	132	83.1	25.6	1 / 0.4%	NA
If Fellow eye 4	+	92	87.3	20.6	5 / 3.2%	31 / 20%
If Fellow eye CNV Overall	NA	128	83.3	23.3	5 / 2%	25 / 10%
If Fellow eye CNV	-	80	81.6	23.8	1 / 0.6%	12 / 8%
If Fellow eye CNV	+	48	86	22.3	4 / 4.5%	13/ 15%

AREDS Cat = age related eye disease study simplified disease categorization scale. RPD = reticular pseudodrusen. GA = geographic atrophy. No. = number. Avg. = average. FU = length of postoperative follow-up, in months. CNV = choroidal neovascularization. Annualized rate = number of new CNV observed divided by months of follow up times 12. Unilateral = Expected = rate of new CNV for this category (if applicable, as RPD not identified) estimated from the AREDS. As the AREDS did not distinguish geographic atrophy (10% of advanced cases) from CNV (90% of advanced cases) in the outcome of category 3 eyes, the expected CNV rate from the AREDS is arrived at by multiplying the incidence of advanced AMD x 0.9. NA = not applicable as RPD not identified in the AREDS.

* For AREDS categories 2–3, assumes fellow eye is category 3 or better.

** Estimated for eyes with RPD by multiplying AREDS rate x 2 in eyes without fellow eye CNV. For eyes with fellow eye CNV, CATT (Comparative of Age-related macular degeneration Treatment Trial) data accounting for RPD is used. None of these new CNV incidence estimates adjust for patient age; median age this study 84 years; AREDS 69 years; CATT study 79 years.

The average AREDS category for SDM treated eyes was 3.0, and 3.2 for fellow eyes. In the eyes developing new CNV after SDM, the average AREDS category was 3.1 in the treated eye and 3.7 in the fellow eye. Following SDM, no eyes with AREDS category 1 or 2 developed a new CNV. No new CNV developed in SDM treated patients under 70, or over 90 years of age. (Figs 1 and 2) (Tables 1–5)

RPD were present in 214 eyes (39%) with dry AMD prior to treatment. The average age of eyes with RPD was 85 years, while those without RPD averaged 80 years. Of the 9 eyes developing new CNV after SDM treatment, 8 (89%) occurred in eyes with RPD (3.7%; annualized rate 2.0%). All 3 patients with bilateral dry AMD who developed a new CNV also had bilateral RPD. Twenty treated patients were active smokers. None developed CNV after SDM treatment. 308 eyes (56%) were in patients with systemic hypertension. Two developed a new CNV following treatment (0.65%; annualized rate 0.37%). Only RPD (p = 0.01) and systemic hypertension (p = 0.048) were associated with an increased incidence of new CNV following SDM. CNV in the fellow eye did not reach significance for this association (p = 0.054). (Tables 1–5)

### Morphologic effects of SDM treatment

Although not a study endpoint, no changes in macular drusen or other morphologic features were noted in the study population after SDM.

### Adverse treatment effects

There were no adverse treatment effects, including laser-induced retinal damage or treatment-associated visual loss.

### Untreated eyes

19 patients (27 eyes, 14 with fellow eye CNV), aged 71–99 years (92 avg.) were not treated for non-ophthalmic reasons. Thus, these eyes do not represent controls. Four of these eyes (15%; 5.5% annualized) developed new CNV (follow up 19–43 months, avg. 33). Another 10 patients (16 eyes; 6 with fellow eye CNV) aged 56–88 years (avg.76), declined treatment. None developed a new CNV (follow up 15–43 months, avg. 34). Thus, 4/43 (9.3%; 3.4% annualized) of untreated eyes developed a new CNV in the study period.

Combining all untreated eyes, the beginning, ending, and mean change in chart VAs were 0.34 (0.26); 0.6 (0.44) and 0.29 (0.39) logMAR units, worsened.

## Discussion

All patients reported in the current study were managed according to the current standard of care for dry AMD. [13] This included the recommendation of AREDS supplements and Amsler grid self-monitoring, dietary and smoking cessation counseling, and periodic clinical examination. In addition, they were offered and elected SDM. SDM has been shown to be effective in all reported applications without adverse treatment effects. The only effects of SDM are therapeutic. [24–26, 31, 32, 39–46] *In vivo* and *in vitro* studies demonstrate that SDM/sublethal retinal laser elicits retinal proteomic and systemic immunologic effects described as “protective” and “restorative” to the retina. [15–38] In clinical study, SDM has been found to improve retinal function by electrophysiology, and visual function by microperimetry and contrast visual acuity in dry AMD, inherited retinopathies, and open angle glaucoma. [41–43] In principle, by safely improving retinal function—and thus health—SDM should slow the progression of dry AMD, and thereby reduce the risk of visual loss. [21–24, 32–38, 41, 47, 48] Our finding of a very low incidence of new CNV in dry AMD following SDM may reflect such processes, and lend support to measures of retinal function as surrogate indicators of disease progression. [47, 48]

The Age-Related Eye Disease Study (AREDS) showed that long-term oral antioxidant vitamin therapy combined with zinc could reduce the risk of advanced AMD and visual loss in dry AMD. [9, 10, 13] AREDS subjects were recruited from retinal subspecialty practices across the US and categorized according to AMD morphology at study entry. For eyes receiving vitamin and zinc therapy with non-advanced AMD (defined as the absence of CNV, subfoveal geographic atrophy, or visual loss due to advanced AMD in either eye) the 5-year estimated probability of developing advanced AMD was reduced 25%, from 28% to 21%. For eyes with advanced AMD in the fellow eye, the 5-year probability of advanced AMD was 43%. The primary benefit from treatment was reduction in the incidence of CNV. There was no effect on progression of drusen or geographic atrophy. [9, 10, 13]

Age is the most important risk factor for the presence, severity, and risk of vision loss from AMD. [1–14, 49–56] In the current study, no eye under 70 years or over 90 developed a new CNV. However, these groups were small. Of the 9 new CNV that developed, one patient was 73, two were 75 to 80; the remaining 6 were between 80 and 90 years of age. (Tables 1–4) The limitations of retrospective studies such as this, and the heterogeneity of prior AMD natural history studies, make comparisons problematic. However, it is important to try to bring some broad perspective to the findings we report. In the AREDS (with a median age of 69 years, 15 years younger than present study) the incidence of new CNV was approximately 4% per year in anti-oxidant treated eyes, with the annual rate of disease progression constant over the AREDS study period. [9] Thus, without adjusting for age of any of the other substantially higher risk factors present in the current study, the incidence of new CNV was approximately 80% lower than would be expected compared to anti-oxidant treated eyes in the AREDS. The Beaver Dam Eye study found a 3-times greater incidence of AMD in eyes 75 years or more, compared to those under 75. [3] Jonasson, et al., found the incidence of advanced AMD in patients 85 years or older to be 10 times greater than patients under 75. [57] Applying these findings to adjust only for age, and ignoring all other risk factors, the estimated range for the expected incidence of new CNV in the current study, absent SDM treatment, would be between 12% to 40% per year. Compared to these estimates, the observed rate of new CNV following SDM (0.87%) in the currently study is 93–98% lower. These differences in observed vs. estimates of expected new CNV were observed across all morphologic subgroups, and occurred over and above the effects of antioxidant treatment, without adverse treatment effects. [9, 10, 13, 52, 55] (Table 5)

Increased number, size and density of macular drusen have been associated with an increased risk of developing CNV in eyes with dry AMD. [2–13, 53–58] Macular photocoagulation has been noted to cause local disappearance of drusen, and has thus been investigated as prophylaxis against AMD progression. [51] A recent meta-analysis of 11 randomized studies using laser to reduce drusen, including 2159 patients (3580 eyes) followed as long as 2 years, found that while photocoagulation could reduce drusen and improve visual acuity, it did not reduce the incidence of new CNV (avg. 10% over 3 years, or 3.3%/year). [53] Without any notable change in macular morphology or drusen reduction following SDM, the current study found a lower incidence of new CNV in eyes with bilateral dry AMD (0.49%/year). This is despite higher risk factors in the current study than in the studies included in the Cochrane meta-analysis, principally of advanced age (current study median 84 years; Cochrane meta analysis median 71 years). [51] These findings suggest that improvements in retinal physiologic function may provide protection against CNV in AMD. [41, 46] (Table 6)

**Table 6:** Incidence of new CNV in dry AMD at various time points^1^.

Study for comparison		Months of follow up^2^		
		6	12	24
Current^1^^,^^2^	Cummulative^3^	2/547 (0.37%)	5/446 (1.1%)	7/244 (2.9%)
	Interval^4^	2/547 (0.37%)	3/444 (0.68%)	2/239 (0.83%)
	CCPY^5^	0.73%	0.58%	0.84%
AREDS^6^	Cummulative^3^	2%	4%	8%
	Interval^4^	2%	4%	4%
Virgili^7^	Cummulative^3^	2.1%	4.2%	8.3%
	Interval^4^	2.1%	4.2%	4.2%

^1^. Observed incidences, not adjusted for risk factor differences between studies, including age (current study median 84 years; AREDS 69 years; Virgili 71 years). [9, 10]

^2^. For current study, from date of first SDM treatment.

^3^. Cumulative = number of all new CNV events since study inception thru the time point, divided by number of eyes completing follow-up at each time point.

^4^. Interval = number of new CNV events occurring within the interval, divided by number of eyes at risk.

^5^. Cumulative new CNV in cases per person-year x 100

^6^. Estimate based on cumulative 21% of advanced AMD developing in treated eyes at 5 years, 90% representing new CNV. (Incidence of new advanced AMD in AREDS found to be constant over 5 years.) [9]

^7^. Incidence of new CNV in untreated control eyes of patients with bilateral drusen reported in the Cochrane meta analysis of studies of laser for drusen. [53] (No risk reduction was noted in treated eyes.) 6 and 12 month incidence estimates based on 24 month incidence of 8.3%, assuming a constant rate of new CNV as per the AREDS. [9]

Recognition of RPD as a distinct clinical entity and independent risk factor for CNV in AMD is relatively recent, and thus is not accounted for in most prior natural history studies, including the AREDS. [1–13, 55–59] In the current study, RPD were the single most important morphologic factor predisposing to conversion to neovascular AMD (p = 0.01). (Table 3) As with other manifestations of AMD, this study found the presence of RPD to parallel patient age, as the average age of eyes with RPD was higher (avg. 85 years) than those without RPD (avg. 80 years) in every AREDS category. (Tables 2 and 5) The CATT study found the presence of RPD to double the risk of new CNV in eyes with fellow eye CNV, compared to eyes without RPD, across all AREDS categories. [53] In the CATT study, the 2-year incidence of new CNV in eyes with RPD and CNV in the fellow eye was 28.7% for AREDS cat 2; 38.7% in AREDS category 3; and 50.5% in AREDS category 4. [54] In the current study, eyes with RPD in the SDM treated eye and a fellow eye CNV also had the highest incidence of new CNV (4/48 or 8.3% over 22 months; or 4.5%/ year). However, this non-age adjusted CNV incidence is markedly lower than that observed for any AREDS category in the CATT. [55] (Tables 4 and 5)

In a recent report, Dias and associates describe the natural history of dry AMD in a group of eyes with fellow eye CNV, evaluated by OCT angiography (OCTA). [59] At presentation, in 110 eyes with intermediate and 50 eyes with geographic atrophy, they found subclinical CNV in 23/160 (14.4%) eyes, while another 6 eyes (3.4%) developed new CNV later, for a total of 29/160 (18%) with subclinical CNV. With at least one follow up visit on 134 eyes, the Kaplan-Meier cumulative incidence of new exudative CNV at 12 months was found to be 6.8% overall; 21% in eyes with non-exudative CNV at presentation, and 3.6% if no subclinical CNV was detected by OCTA at presentation. As a new technology, OCTA was not available to the current study. Although it is difficult to compare, the Dias group is not dissimilar to ours with respect to age (80 vs. 84 years) and AMD severity. Because our group is older, with unusually high risk factors for CNV, it is reasonable to expect that subclinical CNV in our study population was at least as prevalent as in the Dias group (18%). Despite this, our overall annualized incidence of new clinically exudative CNV was lower (2.1% vs. 6.8%). If we imagine the unlikely prospect that our group had no subclinical CNV at study entry, our annualized incidence of new exudative CNV remains lower the Dias group without subclinical CNV (2.1% vs. 3.6%). Dias did not report the prevalence of RPD. In the present study, RPD was the most significant risk factor for developing new clinically active CNV, and strongly associated with age (39% overall; those without RPD averaging 80 years, similar to the Dias group; while those with RPD averaged 85 years of age, close to our median age of 84). A higher prevalence of RPD (doubling the risk of new clinical CNV) in our group compared to Dias, which is thus likely, would suggest a greater advantage from SDM. [55] Thus, comparison with Dias suggests that SDM may confer protection against clinically exudative CNV, even in eyes with pre-existing subclinical CNV. [59] Further study is necessary in this regard.

In eyes with bilateral RPD (AREDS categories 3 and 4) and no pre-existing CNV in the fellow eye, 4/166 (2.4%; 1.3% per year) developed a new CNV after SDM. In the AREDS, patients with bilateral category 3 and 4 dry AMD and a median age of 69 years progressed to advanced AMD (90% due to CNV) at a rate of over 5.2% per year. [10] In the current study, 30 eyes were 70 years or less. None had RPD. If we assume that RPD were not a significant factor in the AREDS based on age; and if, according to the CATT, the presence of RPD doubles incidence of new CNV in all AREDS categories, then the estimated incidence of new CNV of eyes with bilateral RPD and no prior CNV would be at least 10% per year—nearly 10 times the rate observed in the current study following SDM (1.3% per year). [1–13, 55] (Tables 4 and 5)

The findings we report are consistent with the current understanding of the action of SDM on the retina, which is described as a “reset” phenomenon. [33, 41] The initiating step in the response to SDM appears to be sublethal (“low-intensity”) thermal activation of RPE HSPs. [15–41] This has been confirmed by both *in vitro* and *in vivo* studies. [15–38] Activated of HSPs have a multitude of actions directed toward preserving the cell in the face of acute stressors and protecting it against subsequent insults (“conditioning”). HSP functions include repair of misfolded and aggregated intracellular proteins, and enhancement of mitochondrial function and cell metabolism, normalizing RPE function (homeotrophy), cytokine expression and retinal autoregulation, and are anti-apoptotic and anti-inflammatory. [15–37] The actions of HSPs are catalytic and nonspecific, largely independent of the cause of dysfunction; hence the allusion to the familiar “reset” function electronic devices. [33] HSPs are not activated by the slowly developing subacute dysfunctions that characterize chronic progressive disease, such as senescence. Thus, failure or dysfunction of the HSP system itself is a common finding and contributor to chronic diseases of all types. [58–63] By delivering a sudden, severe, but sublethal shock to the RPE (via a temperature change of approximately 10° at the rate of 100,000° C / second), SDM activates HSP-initiated reparative processes that have not been engaged by the underlying chronic disease process; in this case, AMD. [15–37, 58–63] In addition to normalizing retinal proteomics, the systemically immunomodulating effects of HSP activation have been demonstrated following SDM treatment in a murine model. [20, 23, 37] In this study, Caballero, Kent and associates showed that SDM treatment of one eye resulted in recruitment of bone-marrow derived cells to the retina and RPE of both eyes. [37] The constellation of such SDM-elicited HSP-initiated effects could account for the observations in the current study.

Therapeutic improvements in retinal function, and thus retinal health, that may impede development of macular neovascularization might also inhibit development and/or progression of geographic atrophy. As geographic atrophy was not a study endpoint, we cannot comment. However, the long-term stability of visual acuity in the patients we report suggests development and / or progression of GA following SDM was infrequent.

Standard weaknesses of retrospective studies include lack of treatment controls, patient selection bias, incomplete data, loss of follow up, a well-defined outcome measure, and lack of uniformity of both subjects and treatment. [64, 65] Each is minimized in the current study. We report a large number of well-documented subjects with exceptionally high risk factors for the primary treatment outcome. As a retrospective review, there are no treatment controls. However, all treatment-eligible eyes are accounted for. Treatment was offered to 95% of eligible patients and elected by 97% of those, minimizing potential selection bias. Treatment was identical in all patients with regard to technique, and laser parameters. Follow-up is long (up to 9 years; avg. 22 months) and complete (98%). The primary outcome measure of CNV is standard, binary, objective and documentable.

Despite these strengths, as a retrospective observational cohort pilot study, there are questions this study cannot answer. [64, 65] Comprehensive analysis is the province of prospective randomized clinical trials (RCTs). Each type of study has a distinctly different and important investigative role: pilot studies emphasizing on exploration and discovery; RTCs, confirmation. Answers are only as good as the questions asked. Thus, the best RCTs rely heavily on prior pilot studies to inform their design, and to justify the time and expense required to organize and perform them. The findings of such pilot studies must be, first of all, novel and compelling. Secondly, such pilot studies must be of sufficient size and quality to suggest that the key findings will be borne out in more rigorous study. We believe the current study is such a pilot study, deserving of further study in an RCT. [64, 65] If confirmed, panmacular SDM could offer an important, new, and effective treatment for patients hoping to minimize their risk of visual loss due to AMD. [66–68]
